# Polycondensation of a Perylene Bisimide Derivative and L-Malic Acid as Water-Soluble Conjugates for Fluorescent Labeling of Live Mammalian Cells

**DOI:** 10.3390/polym10050559

**Published:** 2018-05-22

**Authors:** Ji He, Huixin Chen, Yanjia Guo, Liang Wang, Lingli Zhu, H. Enis Karahan, Yuan Chen

**Affiliations:** 1 Tianjin Key Laboratory of Organic Solar Cells and Photochemical Conversion, School of Chemistry and Chemical Engineering, Tianjin University of Technology, Tianjin 300384, China; heji0724@126.com (J.H.); 13820608267@163.com (H.C.); hxhg19413@163.com (Y.G.); tjlghxhghj@163.com (L.Z.); 2 School of Chemical and Biomedical Engineering, Nanyang Technological University, 62 Nanyang Drive, Singapore 637459, Singapore; hekarahan@ntu.edu.sg; 3 School of Chemical and Biomolecular Engineering, The University of Sydney, Sydney 2006, Australia; yuan.chen@sydney.edu.au

**Keywords:** facile synthesis, perylene bisimide, poly(α,β-malic acid), cell labeling, fluorescence

## Abstract

Based on simple mixing and polymerization of a hydroxyl-containing derivative of perylene bisimide (PBI) and l-malic acid, here, we demonstrate a new type of dye-polymer conjugate, PBI-poly(α,β-malic acid) (PBI–PMA). Benefiting from the excellent water-solubility of weak polyanionic PMA structure and the high fluorescence of PBI, the PBI-PMA conjugates readily dissolve in water, displaying strong pH-dependent fluorescence with the highest intensity at pH 6. Due to the excellent biocompatibility of PMA, those conjugates showed low cytotoxicity on L929 cells. Using L929 and HeLa cells, we also confirmed that the PBI-PMA-labeled cells display intense fluorescence. Overall, the PBI-PMA conjugate demonstrates high potential as a cell labeling agent with its synthesis ease, good solubility in aqueous medium, low cytotoxicity, and high fluorescence.

## 1. Introduction

Perylene bisimide (PBI) has high potential as a fluorescent label for cell staining thanks to its high fluorescence quantum yield, long band-gap, and good fluorescence stability [1,2,3]. However, the difficulty comes from the strong intermolecular π–π interactions of PBIs’ large aromatic core, which causes water insolubility [4,5]. Several studies have focused on introducing ionic groups or attaching water-soluble polymers to its bay or imide positions [6,7]. For example, Müllen et al. introduced ionic aromatic systems, such as phenoxy substituents with sulfuric acid residues, pyridinium salts, or quaternary ammonium salts, at its bay positions to suppress aggregation in water [8]. Wüerthner et al. targeted imide positions instead and synthesized water-soluble PBI derivatives with terminally linked polyglycerol dendrons [9]. Alternatively, Neoh et al. linked linear water-soluble polymers to PBI with poly[poly(ethylene glycol) methyl ether methacrylate] [10]. Our group also previously synthesized water-soluble PBI-incorporated polyurethanes [11]. Although those water-soluble PBIs have commonly demonstrated good solubility and fluorescence, their relatively tedious synthesis methods pose a disadvantage. Also, the water-soluble polymer systems so far tested are usually not biodegradable, or their degradation products are not totally safe.

Poly(α,β-malic acid) (PMA) is a polyester well-known with high water-solubility, biocompatibility, biodegradability, and bioresorbability [12,13]. Importantly, the final degradation product of PMA; i.e., l-malic acid, is an intermediary product of the tricarboxylic acid cycle of carbohydrate metabolism. In accordance, PMA and several PMA-based polymers have been reported in the form of hydrogels [14], tissue engineering scaffolds [15], and drug carriers [16] for biomedical applications. Although PMA is available from natural sources like bacteria, PMA polymers are mostly synthesized via the polycondensation method [17,18,19]. The polycondensation of PMA, based on the dehydration reaction between hydroxyl and carboxyl groups of l-malic acid, is a relatively simple chemistry. However, the ratio between hydroxyl and carboxyl groups in the malic acid is 1-to-2, which is a hindrance to obtaining high molecular weight PMA. Thus, introducing other compounds having two or more hydroxyl groups into the polycondensation system is beneficial for improving the molecular weight of PMA [20].

In this work, we conjugated PBI with PMA to improve PBI’s water-solubility and biocompatibility. PBI–PMA polymers were synthesized via the polycondensation of PBI–OH and l-malic acid. PBI–OH with two hydroxyl groups plays a dual role as both the fluorescence source and the polymer chain extender, while L-malic acid serves as the water-soluble segment. We showed that varying the PBI–OH content and reaction conditions (i.e., temperature and time) help to increase the average molecular weight of PBI–PMA. We further evaluated the applicability of PBI–PMA in biomedical applications as a fluorescent agent by a cellular toxicity test, MTT assay, and cell labeling experiments.

## 2. Materials and Methods

### 2.1. Materials

Perylene-3,4,9,10-tetracarboxylic dianhydride, diglycolamine, and l-malic acid were procured from Energy Chemical Co., Ltd. (Shanghai, China). Other reagents and all solvents were obtained from Tianjin Guangfu Fine Chemical Research Institute (Tianjin, China). Solvents were analytical grade, and all chemicals were used as received without further purification. *N*,*N’*-bis(2-(2-hydroxyethoxy)ethyl) perylene-3,4,9,10-tetracarboxylic acid bisimide (PBI–OH) were synthesized following a previously reported method [21].

### 2.2. Synthesis of PBI-PMA

Ten different PBI-PMA samples were prepared in total as three groups varying PBI-OH feeding ratio (Group A), temperature (Group B), and time (Group C). A typical reaction procedure is as follows. First, 9.70 g of L-malic acid (0.072 mol) and 0.30 g of PBI–OH (0.00053 mol) were weighted in a reaction bottle and purged with pure N_2_ gas. Next, their mixture was stirred at 120 °C for 48 h under N_2_ atmosphere using vacuum (1 mm/Hg) to remove water that forms during the polycondensation reaction. After dissolving the crude product in 30 mL of THF, the resulting mixture was first filtered through a short silica gel column to remove the unreacted PBI–OH, which is insoluble in THF. Then, solvent extraction was made by adding 100 mL diethyl ether and 100 mL petroleum ether on THF phase to remove excess monomer, l-malic acid. Obtained polymer precipitate was eventually dried under vacuum, yielding PB–PMA products with dark red color.

### 2.3. Material Characterizations

^1^H nuclear magnetic resonance (NMR) spectra of synthesized polymer conjugates were recorded on a 400 MHz NMR spectrometer (AV-400, Bruker, Bremen, Germany). To determine the molecular weight of the PBI–PMA products, a gel permeation chromatography (GPC) system (Waters 2414, Waters, ‎Milford, MA, USA) was employed. For GPC tests, 2.5 mg/mL PMA samples dissolved in tetrahydrofuran (THF) were used. And, the eluent flow rate and temperature of were set as 1.0 mL/min and 35 °C, respectively. The optical properties of PBI-PMA were characterized in both organic solvent and water by ultraviolet/visible (UV/Vis) (Hitachi 3900, Tokyo, Japan) and fluorescence spectroscopies (Hitachi F4500).

### 2.4. Cell Cytotoxicity Test

L929 cells were employed as a cellular model for checking the mammalian cytotoxicity of PBI-PMA. Prior to the tests, the cell culture media supplemented with various PBI-PMA concentrations (0.05, 0.10, 0.20, 0.40, 0.80, 1.60, and 3.20 mg·mL^−1^) were prepared. The L929 cells were then routinely cultured in 96-well plates with their initial density of ~1 × 10^4^ cells per well for 12 h. Afterward, the medium in each well was replaced with the PBI-PMA-containing medium. The cells were further cultured in these media for another 24 or 48 h. Following the protocol we have reported before [11], the cellular cytotoxicity test was then performed on each well by examining the reduction product of the MTT reagent (3-[4,5-dimethyl-thiazol-2-yl]-2,5-diphenyltetrazolium bromide). For the optical detection of formed formazan crystals, 560-nm measurements were made on a multiplate reader (Tecan GENios, Bern, Switzerland). The cell viabilities for each PBI-PMA concentration was then calculated as the percentages relative to that obtained in the control group in which the cells were cultured without PBI–PMA. The two-sample *t*-test was used to analyze the data from PBI–PMA groups and the control group. The *p*-values less than 0.05 (*p* < 0.05) were considered statistically significant.

### 2.5. Cell Labeling and Confocal Imaging

For the cell labeling tests, L929 cells and HeLa cells were first cultured in glass-bottom plates for 24 h. Then, the cells were subsequently cultured in PBI–PMA-containing media (0.10 mg·mL^−1^) for another 40 min. Then, the cells were washed three times with phosphate buffer saline (pH 7.4) to remove the non-internalized dye-polymer conjugates remaining in media and loosely attached to the cellular membranes. Then, confocal laser scanning microscopy (CLSM) was studied to observe the labeled cells on a Nikon A1 microscope system (Tokyo, Japan).

## 3. Results and Discussions

### 3.1. Synthesis of PBI-PMA

**Scheme 1:**
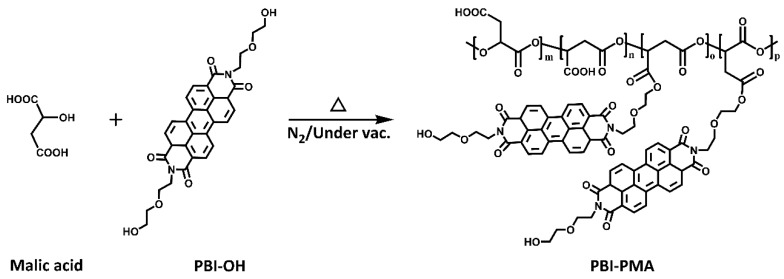
Synthetic production route of PBI-poly(α,β-malic acid) (PBI-PMA). (Note that the PBI–PMA structure shown represents an idealized linear polymer without cross-links. The actual products are expected to be in a partially cross-linked state although we did not observe gelation but instead good solubility).

PBI–PMA was synthesized by the co-polycondensation of l-malic acid and PBI–OH as shown in Scheme 1. Because of its poor solubility in polar environments, PBI–OH cannot be dissolved totally in the melting l-malic acid at the beginning of the co-polycondensation process. Therefore, the mixture appeared as a reddish dispersion first. With the prolongation of the reaction for 10 h, PBI–OH gradually dissolved. And, a red homogeneous solution was obtained, which is mainly composed of the residual melt l-malic acid and the newly formed PBI–PMA. Importantly, PBI–OH with two hydroxyl groups did not induce extreme cross-linking in the resulting PBI–PMA samples. This observation may be attributed to the gradual dissolution and hence controlled feeding of PBI–OH to the reaction media due to its poor solubility. All PBI–PMA samples were observed to be soluble in THF, and their molecular weight could be examined by GPC using THF as the eluent.

To obtain PBI–PMA with higher molecular weight, we studied the effect of three parameters, including the PBI–OH feeding ratio, reaction temperature, and reaction time. The results are summarized in Table 1. When the PBI–OH feeding ratio was increased from 0 to 3 wt %, the molecular weight of PBI–PMA products (Samples 1-A to 1-D) increased from 3.2 to 5.8 kDa. However, PBI–PMA’s molecular weight did not increase further at the feeding ratio of 4 wt % (Sample 1-E). Comparing the Samples 1-A and 1-B, the addition of 1 wt % PBI–OH significantly improves the molecular weight from 3.2 to 4.8 kDa. Previous studies have shown that keeping the amount of hydroxyl and carboxyl functional groups equal is beneficial for obtaining polyesters with high molecular weight [22]. In this work, introducing PBI–OH with two hydroxyl groups into the polycondensation mixture with the carboxyl/hydroxyl ratio of 2/1 helped to increase the proportion of reactive hydroxyl groups. The additional hydroxyl groups serve as the polymer-chain extender to react with the excess carboxyl groups, leading to the formation of PBI–PMA products higher in molecular weight. The reason behind observing similar molecular weights at 3 and 4 wt %, on the other hand, presumably indicates reaching the solubility upper limit of PBI–OH in l-malic acid.

Table 1 also shows that the PBI–PMA production yield increases from 75 to 94% (Samples 2-A to 2-D) with the extension of reaction time from 12 to 60 h. The highest molecular weight is observed at the reaction time of 48 h. Furthermore, Samples 3-A, 1-D, and 3-C compare the effect of the reaction temperatures (110, 120 and 130 °C) on the molecular weight of PBI–PMA. Although the PBI–PMA production yield is higher at 130 °C, the molecular weight reaches the maximum of 5.8 kDa at 120 °C. This outcome can be attributed to the fact that the polycondensation of PBI–PMA is a competitive reaction between the intermolecular and intramolecular dehydration of l-malic acid. While the intermolecular dehydration acts as positive reaction to produce PBI–PMA polymer, the side reaction reduces the molecular weight of PBI–PMA. And, important to note that the intramolecular dehydration of L-malic acid is even severer at high temperatures [23].

**Figure 1:**
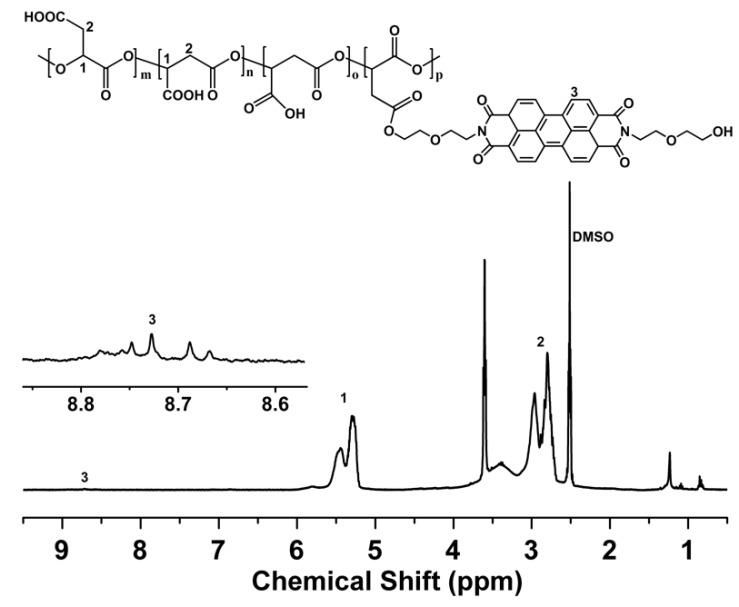
^1^H NMR spectrum of the PBI–PMA Sample 1-D. (Inset figure is the enlarged view of 8th main figure.)

**Figure 2:**
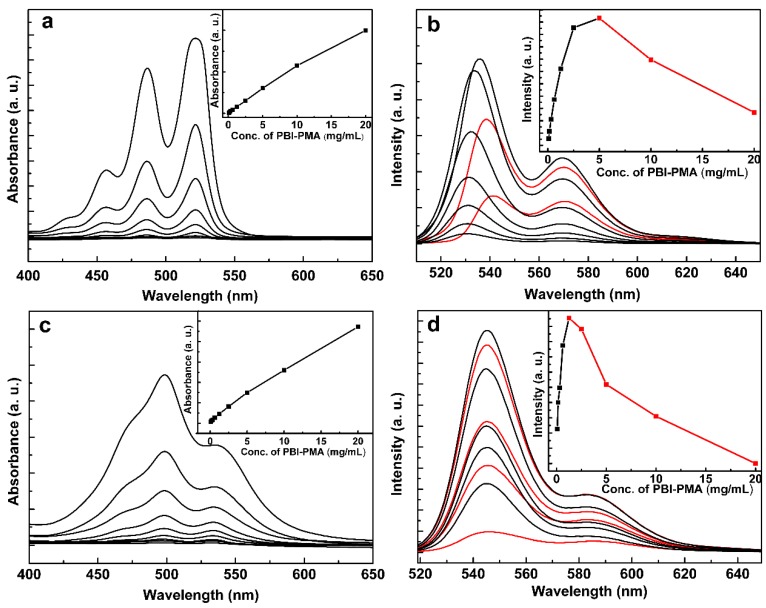
Optical properties of PBI–PMA: (**a**) UV/vis absorption spectra of PBI–PMA in tetrahydrofuran (THF); (**b**) Fluorescence spectra of PBI–PMA in THF; (**c**) UV/vis absorption spectra of PBI–PMA in water; (**d**) Fluorescence spectra of PBI–PMA in water. For all characterizations, the concentrations of PBI–PMA samples are parallel and ranged from 0.78 to 20 mg·mL^−1^.

Since we wanted to test the performance of the sample with the highest molecular weight, we have considered selecting Samples 1-D or 1-E for further studies. Despite the lower PDI of Sample-E, we have selected Sample 1-D for further explorations due to its lower consumption of PBI–OH. As the first step, we have started with studying the chemical properties of Sample 1-D. Figure 1 shows the ^1^H NMR spectrum of Sample 1-D. Two peaks at around 5.4 ppm are attributed to the CH groups in the l-malic acid units. According to the literature [23], the peak at 5.5 ppm is assigned to the α-type l-malic acid unit, while the other peak at 5.3 ppm indicates β-type. The two peaks at around 2.8 ppm can be assigned to the CH_2_ groups in the L-malic acid unit. The signal related to perylene can also be observed at around 8.7 ppm in the enlarged inset of Figure 1, which suggests the successful co-polycondensation of PBI–OH and l-malic acid. Calculated from the integration value of bands (1) and (3) in Figure 2, the PBI content in Sample 1-D is about 2.6 wt %, indicating that over 85% of the PBI-OH precursors introduced have been polymerized with l-malic acid successfully.

### 3.2. UV Absorbance and Fluorescence Properties of PBI–PMA

Concentration-dependent UV/vis and fluorescence spectroscopies were used to study the spectroscopic properties of PBI–PMA in both THF and aqueous solutions. Figure 2a shows that the UV spectra of the PBI–PMA Sample 1-D in THF with its concentration ranging from 0.078 to 20 mg·mL^−1^. There are three peaks at 521, 486, and 455 nm, which are attributed to the 0–0, 0–1, and 0–2 vibronic transitions, respectively [4,24]. The inset of Figure 2a shows that the UV absorbance intensity increased linearly with the increase of polymer concentration. This observation indicates that the aggregation of PBI (through π–π stacking interactions) has been effectively prevented by shielding the perylene cores from assembly via relatively high stereo-hindrance of the large PMA chains. The emission spectra (Figure 2b) presented two major peaks at 525 and 568 nm and one minor peak at around 620 nm, which is in agreement with the previous studies [25,26]. In contrast to their UV spectra, the inset of Figure 2b shows that the fluorescent intensity of PBI–PMA first increases linearly with the concentration from 0.078 to 5.0 mg·mL^−1^, and then it shows a decreasing trend within the concentration range of 5.0 to 20 mg·mL^−1^. At the same time, the fluorescence peak at 530 nm also shifts to 541 nm with the increase of the concentration to 20 mg·mL^−1^. These results suggest fluorescence quenching and π–π stacking of the perylene cores [4,24].

Next, we further studied the concentration-dependent UV/vis and fluorescence spectra of PBI–PMA in the aqueous phase. The results are presented in Figure 2c,d, respectively. Compared to their spectra in THF (organic solvent), the peak patterns shown in Figure 2c in the aqueous solution (Figure 2c) are quite different. The most intense absorption peak comes from the 0–1 vibrational band at 498 nm. The intensity of the 0–0 absorption peak is reduced, and the peak is also slightly shifted towards the red region as the PBI–PMA concentration increased. Similar observations have been made in previous reports focused on the synthesis of some other water-soluble PBI derivatives such as oligomer-tethered PBI and PBI-incorporated poly(glyceryl acrylate) [25,26]. Although the PMA chain enables the water solubility of PBI–PMA, water is not as efficient as THF for dissolving the large aromatic ring of the perylene core. Thus, the aggregation tendency of the perylene cores should be higher in aqueous PBI–PMA than that in THF. The emission spectra given in Figure 2d also show a similar trend as that observed from the PBI–PMA in THF solutions (Figure 2b). The intensity of their fluorescence peaks first increases when the concentration is below 1.25 mg·mL^−1^ and then decreases as the concentration further increasing from 1.25 to 20 mg·mL^−1^ (Figure 2d inset). Notably, the turning point of fluorescent intensity is at 1.25 mg·mL^−1^, which is much lower than the value obtained in the THF solution (5 mg·mL^−1^). This observation also suggests that more aggregation of the perylene cores occurred in water than THF.

### 3.3. Ph–Dependent Fluorescence of PBI–PMA

**Figure 3:**
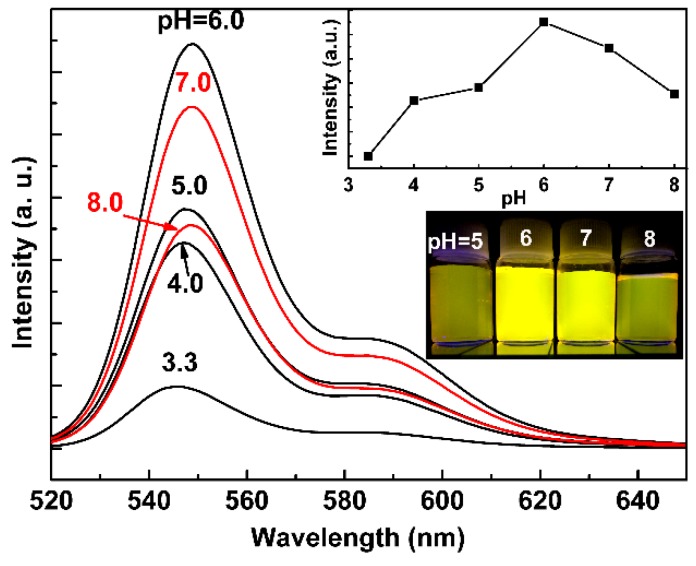
Fluorescence spectra of the PBI–PMA Sample 1-D in the buffers with various pH values with its concentration being 1.25 mg·mL^−1^. (Excitation wavelength is 488 nm. The inset graph and image show the peak maxima of the curves as a function of pH and the photography of samples excited by UV lamp).

PMA with abundant carboxyl groups can be considered as a weak polyanion with pH-dependent solubility in water [27,28]. Therefore, it is useful to study the pH sensitivity of the fluorescence behavior of PBI–PMA before the cell labeling tests. Figure 3 shows the fluorescence spectra of Sample 1-D at the concentration of 1.25 mg·mL^−1^ in several buffer conditions with pH values ranging from 3.3 to 8.0 (Figure 3). The fluorescence intensity of Sample 1-D increases with the increase of the pH until the pH reaches 6.0, and then decreases at higher pH values (pH 7 and 8). Besides, the fluorescence spectrum at pH 8 also exhibits a significant red shift. The high degree of ionization of the carboxyl groups in PBI–PMA can improve its solubility, which might explain the observed fluorescence intensity rise at the pH from 3.3 to 6.0. However, the main component of PBI–PMA, PMA, contains ester groups, which are prone to degradation in aqueous solutions (hydrolysis). And, the degradation of PBI–PMA can be auto-catalyzed by its own carboxyl groups [29]. Further, as we have confirmed with GPC measurements (Supplementary Materials Figure S1), the PBI–PMA degrades significantly at pH 8, which leads to the lower solubility and lower fluorescence intensity of PBI. Overall, the Sample 1-D shows relatively higher fluorescence intensity at pH 6 and 7. The cytoplasmic pH of healthy and cancerous human cells mostly vary around pH 7.0–7.5 and 6.0–7.0, respectively. Since pH 6 and 7 are pretty close to the cytoplasmic pH of cancerous and normal mammalian cells [30,31], the PBI–PMA can be expected to work for a wide range of cell types.

### 3.4. Cytotoxicity and Cell Labeling

Before the cell labeling tests, the cytotoxicity of PBI–PMA should be evaluated. To this end, L929 cells were incubated in the media containing 0.05, 0.10, 0.20, 0.40, 0.80, 1.60, and 3.20 mg·mL^−1^ of PBI–PMA for 24 and 48 h, respectively, and then each of the samples were examined by MTT assay. (The results are shown in Supplementary Materials Figure S2.) After 24 h incubation, a negative correlation was found between the cell viability and the polymer concentration. In detail, as the concentration increases from 0.05 to 3.2 mg·mL^−1^, the viability of L929 cells is reduced from 97.8 to 70.5%. A similar trend is observed after 48 h of incubation. The cellular viabilities are all above 60%, which suggests that most of the cells can sustain their normal metabolic activities after being exposed to the Sample 1-D. The low cytotoxicity can be ascribed to the high content of biocompatible PMA in PMA–PBI conjugates. Water-soluble PMA can also effectively prevent perylene dyes to interact with proteins nonspecifically, shielding the perylene dyes directly attaching with cells and in turn reducing the toxicity of PBI–PMA polymer [32]. Supplementary Materials Figure S2 also shows that the cell viabilities after the 48-h incubation are mostly higher than those after the 24-h incubation. This phenomenon is more notable at the higher concentrations of 0.80, 1.60 and 3.20 mg·mL^−1^. When the incubation time is extended, PMA segments in PBI–PMA may degrade to produce L-malic acid and other oligomers, which can be absorbed and metabolized by cells. This would cause a decrease in the concentration of PBI–PMA in the cell culture media, thus lower the toxicity of high concentration of PBI–PMA in time.

**Figure 4:**
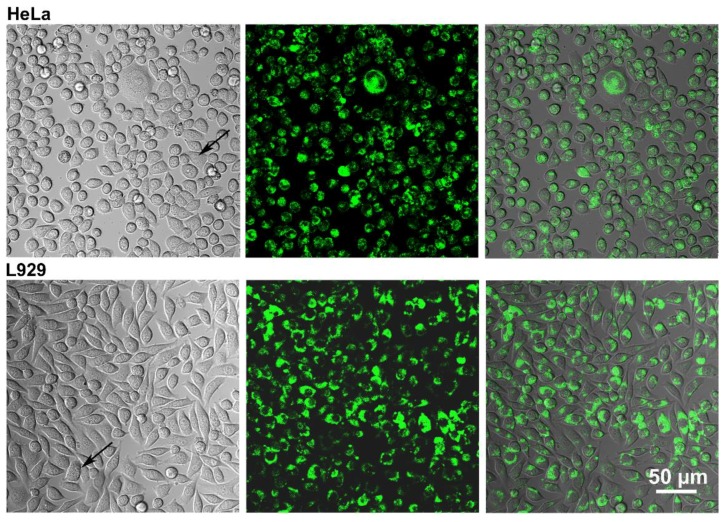
Confocal laser scanning microscopy CLSM images of L929 and HeLa cells stained by 0.10 mg·mL^−1^ PBI-PMA medium for 40 min. (Excitation wavelength is the same as used for fluorescence spectroscopy experiments summarized in Figure 3, 488 nm. All figures share the same scale bar given in bottom right image).

L929 and HeLa cells were used as models of normal and cancerous mammalian tissues, respectively. For both models, the cells were allowed to endocytose Sample 1-D PPBI–PMA found in growth media (0.10 mg·mL^−1^) for 40 min. Then, the brightfield and fluorescence images were taken under the CLSM by focusing at the nuclei of the two cell types. The brightfield images shown in the left column of Figure 4 show expected cellular morphologies, indicating that most of the cells grow well in the media containing PBI–PMA. Strong green fluorescence emitted from the cells shown in the middle column in Figure 4 indicate the fluorescent labeling was successful. The merged images (right column) show that the emission is mainly from the cytoplasm and the area related to cellular nuclei is dark. This observation suggests that the internalized PBI–PMA conjugates accumulate in the cellular cytoplasm but cannot penetrate the nuclei. Also, it is crucial to emphasize that the observation of cell nuclei (as dark spots) in the middle column of Figure 4 can be viewed as an indication of the collection of CLSM images from the middle sections of the cells mostly. In addition, some cells with double nuclei were observed as indicated by arrows in Figure 4. This observation suggests that the cells are still able to divide after the PBI–PMA labeling, which further coincides with the MTT assay results showing low toxicity.

## 4. Conclusions

Insoluble PBI was successfully incorporated into biodegradable PMA via a facile one-step polycondensation reaction. To our knowledge, the PBI-OH has been employed as a polymer chain extender and fluorophore for the first time in this study. The synthesis of PBI–PMA was optimized to obtain higher molecular weight and yield. We found that introducing 3 wt % PBI–OH and performing the reaction at 120 °C for 48 h gives the optimum results, providing PBI–PMA products with the molecular weight value of around 5.8 kDa. PBI–PMA can easily dissolve in water and shows typical UV absorption and fluorescence emission behavior of perylene-based dyes. The fluorescence intensity of PBI–PMA solution is pH-dependent with the strongest fluorescence when the pH is around six, which is a physiologically acceptable condition for both healthy and normal human cells. Further, PBI–PMA shows low toxicity towards L929 cells in the MTT cell viability assay. We have also confirmed the utility of PBI–PMA as fluorescent labels using both L929 and HeLa cell lines. The PBI–PMA-labeled cells not only emitted strong green fluorescence from their cytoplasm but also showed some features of growth morphology. Our results demonstrate that PBI–PMA is a promising cell labeling agent with good water solubility, high fluorescence, and biocompatibility. We plan to benchmark the performance of PBI–PMA with different PBI solubilization strategies in future work.

## Figures and Tables

**Table 1:** Molecular weight, polydispersity index (PDI), and yield of PBI–PMA products synthesized varying reaction time, temperature, and PBI–OH feeding content. (Note that the varied parameters in each experimental group are shown italicized).

Sample		PBI–OH Feeding Ratio (wt %)	Time (h)	Temp. (°C)	Mw (kDa)	PDI (*M*_w_/*M*_n_)	Yield (%)
Group	Code						
1	1-A	*0*	48	120	3.2	1.7	92
	1-B	*1*	48	120	4.8	2.1	93
	1-C	*2*	48	120	5.1	2.0	92
	1-D ^1^	*3*	48	120	5.8	2.2	90
	1-E	*4*	48	120	5.8	1.9	90
2	2-A	3	*12*	120	1.2	1.3	75
	2-B	3	*24*	120	3.1	1.8	86
	2-C	3	*36*	120	4.9	2.0	89
	1-D ^1^	3	*48*	120	5.8	2.2	90
	2-D	3	*60*	120	5.5	2.2	94
3	3-A	3	48	*110*	5.3	2.1	89
	1-D ^1^	3	48	*120*	5.8	2.2	90
	3-B	3	48	*130*	5.2	2.1	95

^1^ The Sample 1-D is shown in all three experimental groups as being the common condition.

## Supplementary Materials

The following are available online at http://www.mdpi.com/2073-4360/10/5/559/s1, Figure S1: GPC analysis of PBI-PMA incubated in PBS for 10 min at pH 6 and 8, respectively; Figure S2: Viability of L929 cells incubated in the media containing 0.05, 0.10, 0.20, 0.40, 0.80, 1.60, and 3.20 mg·mL^−1^ of PBI-PMA for 24 and 48 h.
