# Supplementary material for: Polycondensation of a Perylene Bisimide Derivative and L-Malic Acid as Water-Soluble Conjugates for Fluorescent Labeling of Live Mammalian Cells
Source: Polymers (Basel). 2018 May 22;10(5):559. doi: 10.3390/polym10050559 (PMC6415357; doi:10.3390/polym10050559)
Supplement: Supplementary file 1 [file polymers-10-00559-s001.pdf]

polymers MDPI

Experimental Procedure for Figure S1: Briefly, 50 mg of sample 1-D were dissolved in 40 mL of PBS at pH 6. At the same time, another 50 mg of sample 1-D were dissolved in 40 mL of PBS at pH 8. After 10 minutes incubation at room temperature, these two samples were immediately frozen in a refrigerator to stop the hydrolysis of the ester bonds in the PBI-PMA. Then, the two samples were freeze-dried. The samples was first dissolved in THF and passed through a silica column to remove the PBS salts before running GPC.

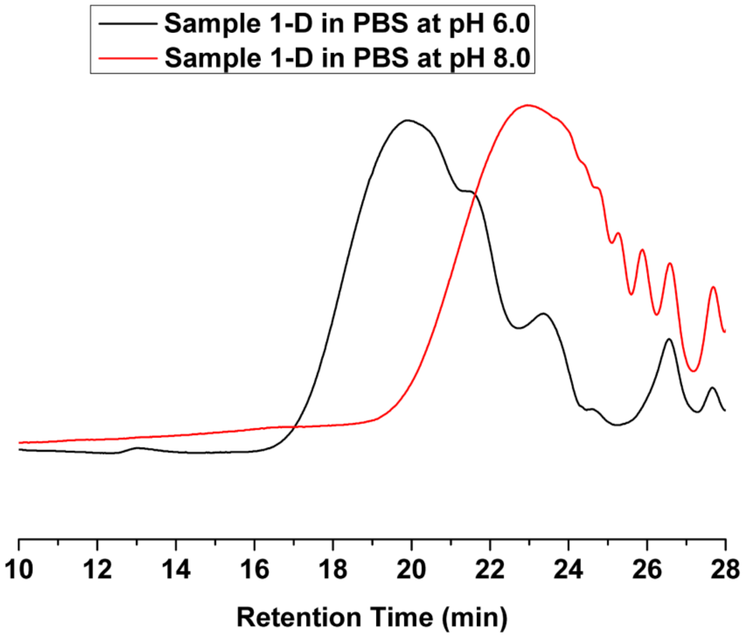

Figure S1. GPC analysis of PBI-PMA incubated in PBS for 10 min at pH 6 and 8, respectively.

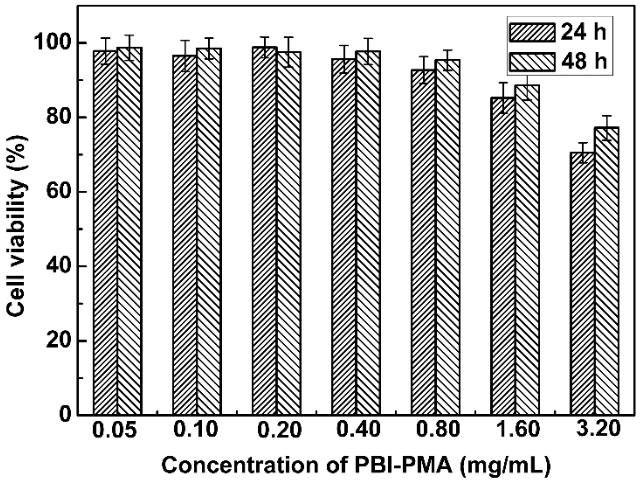

Figure S2. Viability of L929 cells incubated in the media containing 0.05, 0.10, 0.20, 0.40, 0.80, 1.60, and 3.20 mg/mL of PBI-PMA for 24 and 48 h.
